# Altered Localization of p120 Catenin in the Cytoplasm Rather than the Membrane Correlates with Poor Prognosis in Esophageal Squamous Cell Carcinoma

**DOI:** 10.1371/journal.pone.0118645

**Published:** 2015-03-18

**Authors:** Tian Chen, Chen Wang, Fang Wu, Xuebang Zhang, Han Yang, Xia Deng, Qiancheng He, Wenfeng Li, Gang Li

**Affiliations:** 1 Department of Radiotherapy, Ningbo Medical Treatment Center Lihuili Hospital, Ningbo, Zhejiang, P.R. China; 2 Department of Gastroenterology, Taizhou Enze Medical Center Luqiao Hospital, Taizhou, Zhejiang, P.R. China; 3 Department of Gastroenterology, The First Affiliated Hospital of Wenzhou Medical University, Wenzhou, Zhejiang, P.R. China; 4 Department of Chemoradiation Oncology, The First Affiliated Hospital of Wenzhou Medical University, Wenzhou, Zhejiang, P.R. China; 5 Department of General Medicine, The First Affiliated Hospital of Wenzhou Medical University, Wenzhou, Zhejiang, P.R. China; Istituto dei tumori Fondazione Pascale, ITALY

## Abstract

**Background:**

P120 catenin (p120ctn), a regulator of cell adhesion, has previously been found in many malignancies, and suggested a role in invasion, metastasis and survival. The aim of this study was to investigate correlations between altered localization of p120ctn and clinical-pathological characteristics in esophageal squamous cell carcinoma (ESCC).

**Methods:**

Immunohistochemical staining for p120^ctn^ was performed on tissue samples from 118 patients with ESCC. The expression of p120^ctn^ was scored for intensity and cellular localization by Image-pro Plus 6.0. Correlations between immunohistochemical staining of p120^ctn^ and pathological characteristics and clinical prognosis were determined using SPSS 18.0 software.

**Results:**

Membrane expression of p120^ctn^ in ESCCs was lower than that in adjacentnormal esophageal epithelial tissues (P = 0.041), while overall cellular expression of p120^ctn^ was not different between the two tissue types (P = 0.787). Furthermore, neither overall cellular expression nor localized membrane expression was associated with histological and clinical variables. The high ratio of membrane expression to overall cellular expression (M/C) of p120^ctn^ was inversely associated with lymph node invasion (P = 0.001), tumor differentiation (P = 0.012) and advanced tumor stage (P = 0.005); however, it was poorly associated with T stage (P = 0.274). The high M/C ratio of p120^ctn^ was inversely correlated with poor survival; the 5-year OS (overall survival) and the 5-year DFS (disease free survival) for the high M/C ratio group were significantly higher than those of the low M/C ratio group (41.0% vs. 6.7%, P = 0.000; 44.1% vs. 24.9%, P = 0.007). Both the M/C ratio of p120^ctn^ and N status were independent variables for the prediction of overall survival (P = 0.007 and P = 0.027). The M/C of p120^ctn^ predicted a 0.49-fold risk of ESCC death (p = 0.007, 95% CI 0.29–0.83).

**Conclusions:**

The reduced M/C ratio of p120^ctn^ acted as an independent prognostic factor for ESCC patient survival and for the migration and invasive behavior of the disease.

## Introduction

Esophageal carcinoma is the eighth most common malignancy in the world, particularly in Southern and Eastern Africa and Eastern Asia, with a nearly 16-fold variation in incidence internationally. Furthermore, esophageal carcinoma is the sixth leading cause of cancer deaths worldwide [1]. Among all histological sub-types, esophageal squamous cell carcinoma (ESCC) is the most prevalent, accounting for approximately 90% of all esophageal cancers [1–2]. The prognosis of ESCC patients is still poor, with an overall 5-year survival rate of only 10% [3]. Two of the most common prognostic factors of ESCC are an advanced stage at diagnosis and the spread of neoplastic cells into the lymph nodes, and these result in the poor survival of ESCC patients [4–6]. Therefore, the identification of sensitive and representative target genes for the determination of tumor invasion and metastasis is extremely important for treatment and prognosis estimation.

P120ctn, originally identified as a substrate for src kinase, is another member of the catenin family along with α-catenin, β-catenin, and γ-catenin) [7–8]. Like β-catenin and γ-catenin, P120ctn is physically or functionally linked to a wide variety of oncogenes and tumor suppressors, including Src kinases, receptor tyrosine kinases, receptor tyrosine phosphatases, E-cadherin, β-catenin, APC, RhoGTPases, Kaiso, and Wnt signaling effectors. This suggests prominent roles of P120^ctn^ in cell adhesion, motility, morphology, and growth [9]. P120^ctn^ interacts with the cytoplasmic tail of classical cadherins facilitating cadherin clustering to mediate strong cell-cell adhesions [10–11]. P120^ctn^ binds directly to the juxtamembrane domain (JMD) of E-cadherin, unlike β-catenin and γ-catenin, which bind to the catenin-binding domain (CBD) of E-cadherin [12–13]. Interestingly, several reports have indicated that an E-cadherin construct lacking the CBD would retain the adhesive properties of E-cadherin. In contrast, a construct lacking the JMD would not have strong adhesion properties [14–16]. Recent data also indicate that the expression of E-cadherin is dependent on p120^ctn^ function [17]. Therefore, p120^ctn^ probably plays a major role in the maintenance of intercellular adhesion. Moreover, the two domains of p120^ctn^ are dynamically distributed in the membrane and cytoplasm according to their phosphorylation status. Normally, p120^ctn^ is expressed in the membrane and binds to the JMD of E-cadherin, which activates Rho GTPase-Cdc42 and results in the competitive binding of the downstream effector IQGAP1; this maintains the stability of intercellular adhesion. In contrast, when p120^ctn^ is phosphorylated, its expression in the cell membrane and cell-cell junctions is reduced, and its expression in the cytoplasm is increased; this induces Cdc42 inactivation and weakens cell adhesion. Thus, these two cellular distributions of p120^ctn^ can regulate cell adhesion and migration [18–20]. In summary, p120^ctn^ plays an important role in tumor cell adhesion and tumor metastasis, occurrence and development.

Previous in vitro and in vivo studies have shown that the transition of p120^ctn^ from the membrane to the cytoplasm is associated with cell migratory activity, a potentially invasive phenotype and disease progression [21–24]. Furthermore, evidence that reduced or altered expression of p120^ctn^ is related to invasion and metastasis of primary tumors to many organs, such as colon, bladder, esophagus, prostate, lung, breast and biliary tract, is accumulating. Moreover, some research has indicated that p120^ctn^ expression pattern correlates with cancer prognosis [25–33]. To date, no research has thoroughly demonstrated the correlation between p120^ctn^ expression and the clinical prognosis of ESCC patients. Therefore, the present study focused on altered p120^ctn^ expression and localization in 118 cases of primary ESCC using immunohistochemistry. The study also analyzed the relationship between the immunohistochemistry results and different pathological characteristics and clinical prognoses.

## Materials and Methods

### Patients and Tissue Specimens

#### Ethics Statement

The experimental protocol was approved by the Research Ethics Committee of Wenzhou Medical University, Wenzhou, China. All patients provided written informed consent. This study included resection specimens of 118 patients with ESCC, who all underwent esophagectomy without pre-operative treatment between January, 2006 and June, 2013 at the first affiliated hospital of Wenzhou medical university. All specimens were squamous cell carcinoma confirmed by pathological diagnosis. The study group consisted of 111 males and seven females with a median age of 60 years (range 40–80 years). pTNM staging was performed according to the criteria of the TNM classification system of malignant tumors (AJCC, 2002). Patients with one or more local-regional lymph nodes invaded from cancer should be considered as N positive. All the patients underwent radical operation had no distant metastasis in the study before surgery. Table 1 summarizes the histological and clinical variables of these patients.

**Table 1:** Relationship between p120ctn localization and histological and clinical variables.

Variables	patients	membranous p120^ctn^			cellular p120^ctn^			M/C		
		-	+	P value	-	+	P value	-	+	P value
**Age(mean ± SD)**	60.08±9.60			.850			.118			.738
⩾65		22	23		18	27		23	22	
＜65		37	36		40	33		35	38	
**Gender**										.715
Male	111	54	57	.439	54	57	.715	54	57	
Female	7	5	2		4	3		4	3	
**Smoking status**				.398			.072			.596
Smoker	88	42	46		39	49		42	46	
Non-smoker	30	17	13		19	11		16	14	
**Drinking status**				.432			.389			.389
Drinker	101	49	52		48	53		48	53	
Non-drinker	17	10	7		10	7		10	7	
**Histological differentiation**				.046			.903			.012
Well(G1/G2)	82	36	46		40	42		34	48	
Poor(G3/G4)	36	23	13		18	18		24	12	
Tumor location				.309			.500			.313
Upper	17	10	7		8	9		10	7	
Middle	62	27	35		33	29		27	35	
Lower	31	16	15		12	19		15	16	
Double	8	6	2		5	3		6	2	
**T-stage**				.196			.468			.274
Early(T1/T2)	63	28	35		29	34		28	35	
Late2(T3/T4)	55	31	24		29	26		30	25	
**N-stage**				.065			.186			.001
Positive	33	21	12		13	20		24	9	
Negative	85	38	47		45	40		34	51	
**Stage grouping**				.007			.461			.005
Early(I/II)	87	37	50		41	46		36	51	
Advanced(III)	31	22	9		17	14		22	9	

### Immunohistochemistry (IHC)

Both tumor and adjacent normal tissue blocks were cut into 5-μm-thick sections and mounted on glass slides. Immunohistochemical staining for P120^ctn^ was performed with an EnVision method (Da Ko Corporation). In other words, the sections were dewaxed, incubated with methanol containing 30% H_2_O_2_ for 20 min to block endogenous peroxidase activity, immersed in 0.01 mol/L citrate buffer (pH 6.0), heated at 100 in a microwave oven for 20 min, washed three times with distilled water and blocked with 1℅ BSA for 30 min. Then, the sections were incubated overnight at 4 with rabbit polyclonal IgG against p120^ctn^ (H-90, SC-13957, Santa Cruz Corporation, USA) at a 1:150 dilution. A subsequent reaction was carried out using secondary antibodies (Dakocytomation Company, Denmark) at 37 for 30 min. Then, the sections were washed three times with phosphate-buffered saline (PBS) and, subsequently, the staining was visualized by incubating with DAB (Dakocytomation Company, Denmark) for approximately 4 min. The nuclei were lightly counterstained with hematoxylin. No staining was obtained when immune serum or PBS was used instead of primary antibodies, confirming the specificity of each primary antibody. Known immunoreactive sections were used as positive controls.

### Evaluation of immunostaining

The immunoexpression level was assessed by manual counting and was aided by analysis using Image-pro Plus 6.0 (IPP 6.0) as described previously [34]. The measurement parameter was integrated optical density (IOD). The function of irregular automated optical inspection (irregular AOI) was applied by IPP 6.0 software to score and rule out non-target staining of p120^ctn^. Interesting targets included the overall tumor cell, tumor cell membrane and adjacent normal esophageal epithelial tissue. P120^ctn^ expression was determined by counting 1,000 cells in 10 large graticules visible in the microscope. All images analyzed using IPP 6.0 were verified by two pathologists who were blinded to the results of the previous assessments. When there was a disagreement, a consensus was reached by discussion.

### Statistical analysis

The characteristics of the patients were expressed as percentages or means. Chi-square test was used to estimate the relationship between staining patterns of p120ctn and the parameters, such as histological differentiation, T stage and lymph node invasion. For further analysis, automated IOD scores were converted into binomial variables of high versus low or lost expression around the median. The OS was calculated as the period from the first day after surgery to death or the date of the last follow-up. The DFS was defined as the time from the first day after surgery to the day of disease recurrence. The Kaplan-Meier method was used for survival analysis, and differences in survival were estimated using the log rank test. Statistical analyses were performed using the SPSS 18.0 software package for Windows (Chicago, IL, USA). A p value less than 0.05 was considered statistically significant.

## Results

### P120^ctn^ expression in ESCCs and adjacent normal esophageal epithelial tissues determined by immunostaining

**Fig 1:**
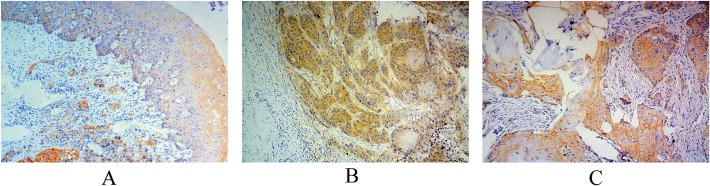
Immunostaining of P120ctn in ESCC and esophageal epithelial tissues. A. Immunohistochemical staining showed p120^ctn^ expressed in tumor cells (the lower left part of the figure) and normal cells (the upper right part of the figure); B. P120^ctn^ was mainly located in tumor cell cytoplasm. C.P120^ctn^ was mainly located in tumor cell cytomembrane.

The level of expression and localization of p120^ctn^ was assessed in 118 ESCC and 72 adjacent normal esophageal epithelial tissue samples (Fig. 1 A-C). For 46 patients, there was a lack of normal epithelial cells in the tissue sections obtained, so adjacent normal esophageal epithelial tissue samples could not be evaluated. The median IOD of the 118 ESCC samples for the entire cell and the membrane were 9863.32 and 3828.41, respectively. The median IOD of the 72 adjacent normal esophageal epithelial tissue samples for the entire cell and membrane were 9973.00 and 5869.00, respectively. These IOD values indicate that the membrane expression of p120^ctn^ in the ESCC samples was significantly lower than in the adjacent normal esophageal epithelial tissues (P = 0.041), while overall cellular expression was not different between the two tissue types (P = 0.787) (Pair test) (Fig. 1A-C). This suggests that membrane localization of p120^ctn^ may distinguish between ESCC and normal esophageal tissue better than overall cellular expression.

### Relationship between p120^ctn^ localization and histological and clinical variables

The median IOD scores for the cellular and membrane localization of p120^ctn^ in 118 tumor sections were used as the high and low cutoff values for p120^ctn^ expression, respectively. We found that neither overall cellular nor membrane expression was significantly associated with histological or clinical variables (Table 1). Taking into account the transition of expression of p120^ctn^ from the membrane to the cytoplasm, which is associated with cell migratory activity and, potentially, an invasive phenotype, the M/C ratio (membranous IOD/cellular IOD) may more clearly indicate relationships between p120^ctn^ expression and histological and clinical variables. For further analysis, the M/C ratios of the 118 patients were used to separate the patients into a high proportion group and a low proportion group according to their median values. The high proportion group was inversely associated with lymph node invasion (P = 0.001), tumor differentiation (P = 0.012) and advanced stage (P = 0.005); however, it was poorly associated with T stage (P = 0.274) (Table 1).

### Relationship between membrane expression of p120^ctn^ and survival

**Fig 2:**
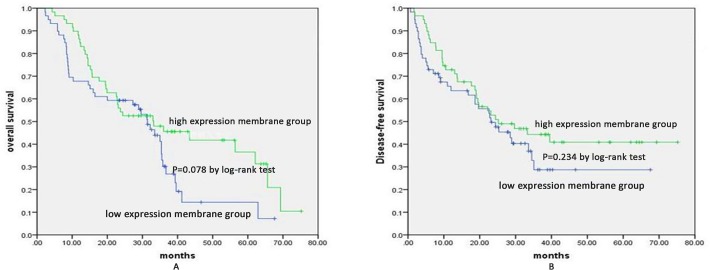
Kaplan-Meier estimates of the OS and DFS according to membrane expression in ESCC patients. A. Kaplan-Meier curves illustrating the OS of the high membrane expression group versus the low membrane expression group. Five-year OS of the high membrane expression group (36.6%) versus the low membrane expression group (14.4%), P = 0.078; B. Kaplan-Meier curves illustrating the DFS of the high membrane expression group versus the low membrane expression group. Five-year DFS of the high membrane expression group (40.9%) versus the low membrane expression group (28.7%), P = 0.234.

**Fig 3:**
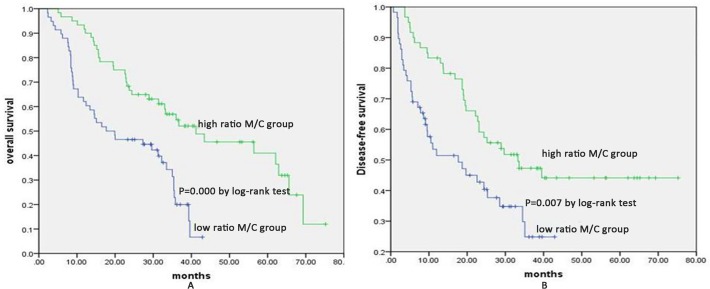
Kaplan-Meier estimates of the OS and DFS according to the rate of membrane expression to whole cellular expression (M/C) in ESCC patients. A. Kaplan-Meier curves illustrating the OS of the high M/C ratio group versus the low M/C ratio group. Five-year OS of the high ratio M/C group was significantly higher compared to that of the low M/C ratio group (41.0% vs. 6.7%, P = 0.000); B. Kaplan-Meier curves illustrating the DFS of the high M/C ratio group versus the low M/C ratio group. Five-year DFS of the high M/C ratio group were significantly higher compared to those of the low M/C ratio group (44.1% vs. 24.9%, P = 0.007).

**Fig 4:**
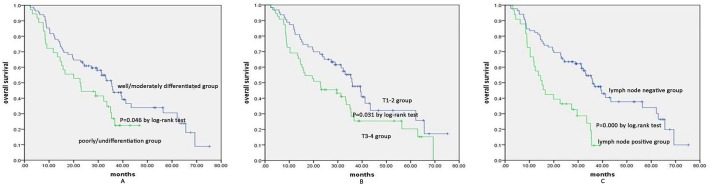
Univariate analysis of tumor grade and T and N status. A.Five-year OS of the well/moderately differentiated group was better than that of the poorly/undifferentiated group (30.5% vs. 22.4%, P = 0.046); B. Five-year OS of the T1–2 group was better than that of the T3–4 group (32.2% vs. 20.3%, P = 0.031); C. Five-year OS of the lymph node negative group was better than that of the lymph node positive group (33.9% vs. 9.5%, P = 0.000).

With a median follow-up of 27.55 months, the 5-year overall survival values were 36.6% and 14.4% for the high membrane expression group and the low membrane expression group, respectively (Fig. 2 A-B). No significant differences in the Kaplan-Meier curve were detected between the two groups (P = 0.078), and there was no significant difference in 5-year DFS between the two groups (P = 0.234) (Fig. 2A-B). Furthermore, a high M/C ratio was correlated with survival benefit, with a 5-year OS of 41.0% and 6.7% for the high M/C ratio group and the low M/C ratio group, respectively (P = 0.000) (Fig. 3A). The 5-year DFS of the high ratio group was also significantly higher than that of the low ratio group (44.1% vs. 24.9%, P = 0.007) (Fig. 3B). The median survival time was 31.9 months for the high M/C ratio patients, which was significantly longer than the survival time for the low M/C ratio patients (18.8 months) (Fig. 3A-B). Furthermore, the univariate analysis revealed that the M/C ratio of p120^ctn^, tumor grade, and T and N status were significantly associated with survival (Fig. 3A and Fig. 4 A-C). The multivariate analysis using the Cox regression model showed that both the M/C ratio of p120^ctn^ and N status were independent variables in predicting overall survival (P = 0.007 and P = 0.027). In the multivariate Cox’s regression analysis adjusted for tumor grade, the M/C of p120^ctn^ with T and N status and M/C of p120^ctn^ with N status alone predicted a 1.84- and 0.49-fold risk of ESCC death, respectively (p = 0.027, 95% CI 1.07–3.18 and p = 0.007, 95% CI 0.29–0.83) (Fig. 4A-C).

## Discussion

In this study, we assessed the expression and localization of p120^ctn^ in a large series of ESCC samples to investigate the potential prognostic value of variations in different clinical parameters. In previous studies, p120^ctn^ was detected in cell-cell boundaries in benign tumors and normal tissues, but in the cytoplasm in carcinoma tissues [35–37]. Our current study also showed that the membranous expression of p120^ctn^ was reduced or lost in ESCC tissues compared with adjacent normal esophageal epithelial tissues (P = 0.041). This study showed that the abnormal expression of p120^ctn^ was closely related to lymph node invasion, advanced stage and tumor differentiation. Our results are similar to those reported for other cancers [27, 28, 33, 36], which indirectly suggests that the absence or decrease of p120^ctn^ in the membrane plays a crucial role in tumorigenesis.

One previous report showed that p120^ctn^ immunoexpression is associated with ESCC. That report by Yvonne Chung et al. [29] showed that membranous expression of p120^ctn^ was related to differentiation and lymph node metastases. Unfortunately, no significant relationship between p120^ctn^ expression and survival was noted. However, aberrant p120ctn expression has been reported as an independent prognostic marker in gastroesophageal adenocarcinoma, breast cancer, bladder cancer, lung cancer and colorectal cancer [27, 28, 31, 36]. It seems reasonable to assume that aberrant p120ctn expression has been reported as an independent prognostic marker in ESCC. We have not found a more appropriate form for predicting prognosis. David I. Bellovin et al. have examined the expression and localization of p120^ctn^ as a consequence of the epithelial to mesenchymal transition (EMT) of highly differentiated colon carcinoma cells (LIM1863 cells). They found that the localization of p120^ctn^ shifts from cell-cell junctions to the cytoplasm, while total p120^ctn^ expression remains stable during EMT [27]. The expression and localization of p120^ctn^ may be altered in the same manner in ESCC. Our study also showed that overall p120^ctn^ expression was not different between ESCCs and adjacent normal esophageal epithelial tissues (P = 0.787). However, the overall expression levels of p120^ctn^ in individual ESCC cells are different. Taking these characteristics into account, the M/C ratio may not only describe membrane expression but also eliminate the individual differences of cells.

In the present study, we selected the IHC method to evaluate p120^ctn^ protein expression in ESCCs mainly because fresh biopsy tissues were unavailable. Although IHC is a semiquantitative technique, it is the most commonly used, simplest, and cheapest protocol in clinical work [38]. The expression of specific location of the molecule examined is scored by IPP software, which is more accurate and objective than counting by an observer. Therefore, cell membrane and overall cell expression are scored with IPP6.0 and are used to calculate M/C ratio. Encouragingly, the M/C ratios observed here were related not only to lymph node invasion, advanced stage and tumor differentiation but also to survival. This further supports p120^ctn^ as a useful prognostic marker for ESCC.

To our knowledge, this was the first study to successfully show that p120^ctn^ expression is correlated with survival in ESCC patients by analyzing the M/C ratio of p120^ctn^ expression. This relationship is not reflected by observer counting [29]. Thus, this method may be a useful scoring method for proteins dyeing experiments, such during EMT, in the future. This study may help popularize this method, which needed to be further verified. In summary, our findings show that p120^ctn^ may be a useful prognostic marker in ESCC and in other tumors. Based on our results, we have developed a new and objective method for scoring p120^ctn^ expression. The M/C ratio of p120^ctn^ acted as an independent prognostic factor for ESCC patients and as a predictor of the migratory and invasive behavior of the disease.
